# CARD9 deficiency with allergic bronchopulmonary aspergillosis (ABPA)-like presentation: a case report

**DOI:** 10.1093/omcr/omad103

**Published:** 2023-10-23

**Authors:** Mazdak Fallahi, Seyed Alireza Mahdaviani, Mohammadreza Shafiei, Soodeh Ghadimi, Nima Rezaei, Christoph Klein, Sophie Strobel, Mahnaz Jamee

**Affiliations:** Pediatric Respiratory Diseases Research Center, National Research Institute of Tuberculosis and Lung Diseases (NRITLD), Shahid Beheshti University of Medical Sciences, Tehran, Iran; Pediatric Respiratory Diseases Research Center, National Research Institute of Tuberculosis and Lung Diseases (NRITLD), Shahid Beheshti University of Medical Sciences, Tehran, Iran; Student Research Committee, School of Medicine, Alborz University of Medical Sciences, Karaj, Iran; Immunology and Allergy Department, Mofid Children’s Hospital, Shahid Beheshti University of Medical Sciences, Tehran, Iran; Research Center for Immunodeficiencies, Pediatrics Center of Excellence, Children’s Medical Center, Tehran University of Medical Sciences, Tehran, Iran; Dr. von Hauner Children's Hospital, Ludwig Maximilians, University Munich, Munich, Germany; Institute of Nutritional Medicine, School of Medicine, Technical University of Munich, Munich, Germany; Laboratory for Pediatric Immunology, Department of Pediatrics, Willem-Alexander Children’s Hospital, Leiden University Medical Center, Leiden, Netherlands

## Abstract

Purpose: We present a patient with CARD9 deficiency and allergic bronchopulmonary aspergillosis (ABPA)-like presentation. Methods: Following medical history taking and routine laboratory investigations, an inborn error of immunity was suspected, and the responsible variant was identified using Whole Exome Sequencing and confirmed by Sanger sequencing. Results: A 14-year-old Iranian female presented with a history of chest pain, productive cough, dyspnea, malaise, and recurrent fever. Imaging by computed tomography (CT scan), chest X-ray (CXR), bronchoscopy, transbronchial lung biopsy (TBLB), and histopathology findings led to a diagnosis of ABPA-like presentation. The genetic study showed an autosomal recessive homozygous mutation in the CARD9 gene. Clinical remission was achieved following the administration of voriconazole, which was continued as prophylaxis. Conclusions: This is the first-time report of a patient with inherited CARD9 deficiency and ABPA-like presentation due to Aspergillus Terrus. This study paves the way to elucidate immunological mechanisms underlying CARD9 deficiency and aspergillosis.

## INTRODUCTION

Caspase Recruitment Domain-containing protein 9 (CARD9) is an intracellular signal transducer protein responsible for transmitting signals from multiple innate immunity recognition receptors, including intracellular NOD and C-type lectin receptors [1, 2]. CARD9 is predominantly expressed by several myeloid cells, including neutrophils, macrophages, and dendritic cells; subsequently, it plays a considerable part in innate immunity [3]. Polymorphisms and mutations in the CARD9 gene appear to contribute to several diseases, including inflammatory bowel disease, autoimmunity, and microbial infection [3].

CARD9 is known to protect against fungal infection by enhancing the pattern recognition receptors. Mutations of CARD9 predispose patients to various fungal infections, mainly due to the decreased peripheral Th17 cells, disrupted proinflammatory cytokines against fungi, and diminished yeast phagocytosis [4, 5]. Loss of CARD9 elicits an Inborn error of immunity (IEI) that is highly susceptible to fungal infections while exhibiting no bacterial, viral, or parasitic infection [1].

Herein, we aim to report a 14-year-old Iranian female harboring a CARD9 mutation with aspergillosis and presenting invasive fungal infection as the initial manifestation of the disorder.

## CASE PRESENTATION

A 14-year-old girl presented to our hospital with a history of allergic rhinitis, left-side chest pain, productive cough, dyspnea, malaise, and recurrent fever from 12 years of age. She was the first child of third-degree consanguineous parents from a village around Bandar-Abbas (a harbor in the south of Iran). Physical examination showed decreased left lung sound, postnasal discharge (PND), and fever.

Family history was unremarkable, except for a history of asthma in her mother. She had taken cefixime and azithromycin for 6 months. Plus, they kept sheep, ducks, and cats at home.

The Chest Computed tomography (CT) a year before reported atelectasis, and collapse in the left lung. A high-resolution CT scan (HRCT) of the lungs, one month prior to being referred to our hospital, showed a ‘finger in glove’ sign, which is suggestive of bronchocele, and suspicious bronchiectasis in the left lung upper lobe.

**Figure 1:**
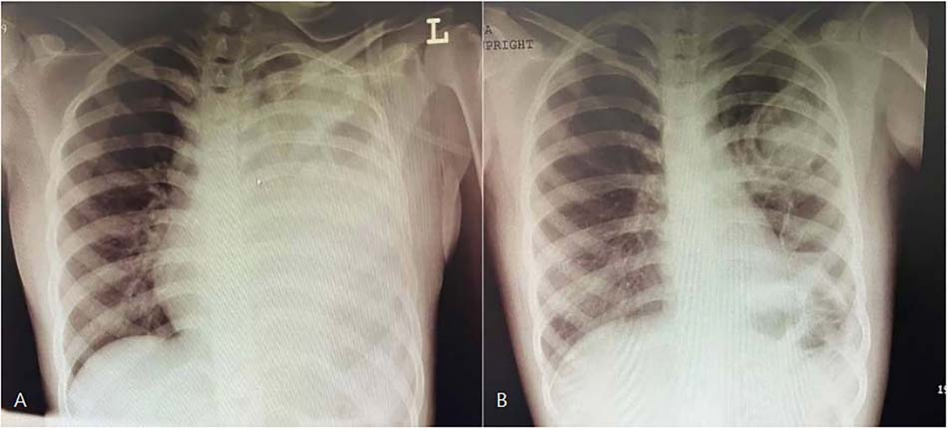
CXR findings in the CARD9 deficient patient (**A**) The first day of patient hospitalization. (**B**) Patient’s CXR after bronchoscopy.

**Figure 2:**
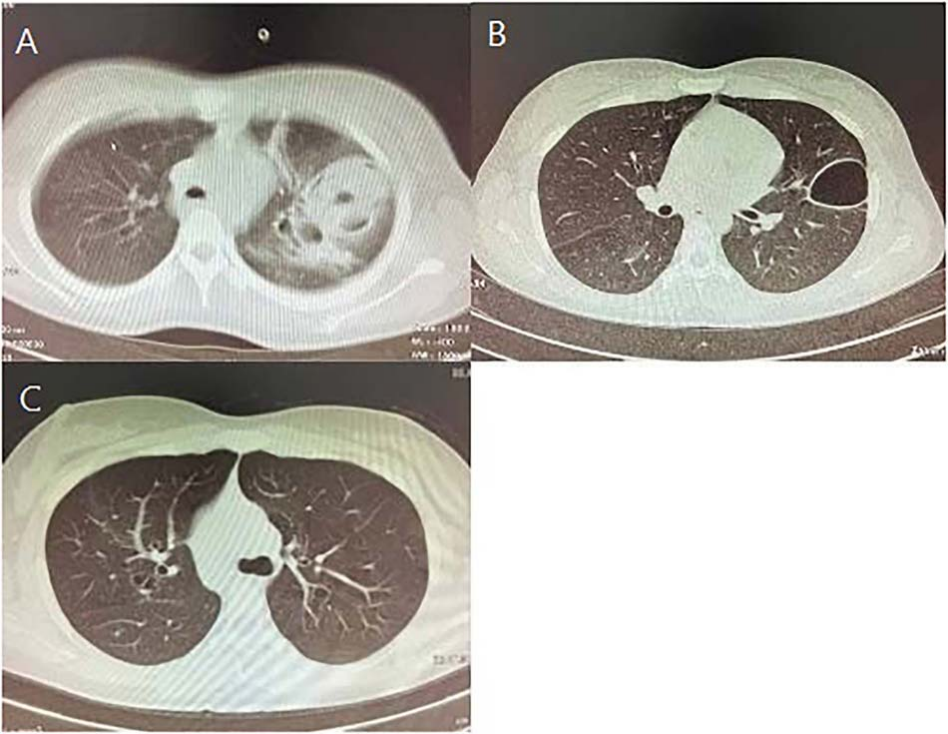
Computed tomography of the chest taken within treatment periods with Voriconazole. (**A**) Right after bronchoscopy (**B**) One month after hospitalization (**C**) 15 month after treatment.

We also performed a chest X-ray (CXR) (Fig. 1) and a chest CT scan (Fig. 2). The CT scan confirmed the cavitary lesion in the left lung, with the opaque view in the left lower lobe, and ground- glass opacity of the right lung. We started broad-spectrum antibiotics for the patient. We referred her to bronchoscopy and transbronchial lung biopsy (TBLB). An obstructed orifice of the left main bronchus (LMB) caused by dense mucosal aggregations and infiltration of lymphocytes and eosinophils through the lamina properia of obtained samples was observed during bronchoscopy and TBLB, respectively. Besides huge amounts of adhesive high mucoid secretion in LMB, patchy erythema of bronchus mucosa was beheld.

Based on the high level of serum total IgE, history of atopy, and chest HRCT findings, we performed a skin prick test for *Aspergillus* and it was positive.

Diagnostic criteria proposed by the International Society for Human and Animal Mycology (ISHAM) for ABPA consists of [6]:

Predisposing conditions (one must be present):

- Asthma
- Cystic fibrosis

Obligatory criteria (both must be present)

- Serum IgE levels against Aspergillus famigatus (>0.35 KU/l) or positive

Aspergillus skin test

- Elevated total serum IgE (typically >1000 IU/Ml) Other criteria (at least two must be present):
- Precipitating serum antibodies to A. fumigatus or elevated *Aspergillus fumigatus* specific IgG levels (>27 mg/l)
- Radiographic pulmonary opacities consistent with ABPA
- Total eosinophil count >500 cells/μl

Our patient fulfilled the criteria such as positive Aspergillus skin test and elevated total serum IgE, increased total eosinophil count, radiographic features such as finger in glove, atelectasis, and bronchiectasis. However, our patient did not men her medical history, there is a history of cough, dyspnea and asthma in her mother, which may indicate undiagnosed asthma. Therefore, we considered this patient as a CARD9 deficient case with ABPA like presentation With an impression of allergic bronchopulmonary aspergillosis (ABPA), we started treatment with voriconazole. After a year, voriconazole was discontinued due to normal chest CT scan reports. However about a month later, she was referred to a hospital in her hometown due to fever, chest pain, and dyspnea, and was found to have a left sided pleural effusion on chest CT scan Table 1. We obtained a sample for Whole Exome Sequencing (WES) and then discharged her with voriconazole after 14 days. We performed WES on the patient using Illumina DNA prep & TWIST comprehensive exome-enrichment library preparation and an Illumina Nova Seq 6000 sequencing platform. The result was an autosomal recessive homozygous CARD9 missense mutation, c.86G>A (p. Arg29His). This variant was classified as likely pathogenic and confirmed by Sanger Sequencing.

**Table 1:** Summary of laboratory findings

Parameter	Result	Sample
WBC (cells/mm^3^)	4900	Blood
Neutrophil (%, cells/mm^3^)	20%, 980	Blood
Lymphocyte (%, cells/mm^3^)	54%, 2646	Blood
Monocyte (%, cells/mm^3^)	11%, 539	Blood
Eosinophil (%, cells/mm^3^)	15%, 735	Blood
CD3+ T cells (% of lymphocytes)	67%, 1772	Blood (immunologic screening)
CD4+ T cells (% of lymphocytes)	37%, 979	Blood (immunologic screening)
CD8+ T cells (% of lymphocytes)	26%, 687	Blood (immunologic screening)
CD19+ B cells (% of lymphocytes)	17%, 449	Blood (immunologic screening)
CD20+ B cells (% of lymphocytes)	17%, 449	Blood (immunologic screening)
CD56+ NK cells (% of lymphocytes)	18%, 476	Blood (immunologic screening)
IgG (mg/dl)	850	Blood (immunologic screening)
IgA (mg/dl)	147	Blood (immunologic screening)
IgM (mg/dl)	126	Blood (immunologic screening)
IgE (IU/ml)	574	Blood (immunologic screening)
ESR (mm/hr)	30	Blood
Fungal culture	Asp. Terreus	Broncho alveolar lavage & TBLB
Pathology	Necroinflammation	Broncho alveolar lavage & TBLB
Galactomannan	8	Broncho alveolar lavage & TBLB
PH	7	Pleural effusion
WBC (cells/mm^3^)	12 700	Pleural effusion
Neutrophil (%)	9%	Pleural effusion
Lymphocyte (%)	91%	Pleural effusion
Glucose (mg/dl)	93	Pleural effusion
Protein (mg/dl)	4.6	Pleural effusion
LDH (mg/dl)	948	Pleural effusion

WBC, white blood cell; Ig, immunoglobulin; ESR, estimated sedimentation rate; LDH, Lactate dehydrogenase.

Currently, she is under prophylaxis with voriconazole, and she has not experienced any recurrence of pulmonary infection.

## DISCUSSION

Although CARD9-deficient patients show high susceptibility to various fungal infections, few cases of infection with *Aspergillus* have been reported until recently [5, 7, 8]. In this study, we reported a 14-year-old Iranian female with CARD9 deficiency who developed several pulmonary complications including atelectasis, bronchiectasis, bronchocele, and pleural effusion caused by *Aspergillus Terreus*.

Aspergillosis is a spectrum of diseases caused by *Aspergillus* ranging from noninvasive complications such as allergic fungal rhinosinusitis and ABPA to Invasive Pulmonary Aspergillosis [9]. The spectrum of clinical manifestations of aspergillosis in patients with CARD9 deficiency is still evolving; the first reported case was of extrapulmonary invasive aspergillosis in a CARD9-deficient patient caused by *Aspergillus fumigatus* [5], and the latest report was of a chronic cutaneous type in a 45-year-old man and cutaneous/pulmonary in CARD9 knock out mice models, caused by *Aspergillus fumigatus* as well [7]. It had been deemed that chronic pulmonary aspergillosis and ABPA occur in patients with mild risk factors and immunocompetent people, respectively, contrary to the CARD9-deficient patients [10]; however, this report about a patient with *Aspergillus Terrus* induced ABPA a chronic pulmonary disease due to *Aspergillus Nomius* and disputes this hypothesis [8]. Therefore, CARD9 deficiency predisposes individuals to a wide spectrum of aspergillosis caused by multiple *Aspergillus* organisms.

Several mechanisms have been proposed as causative agents of impaired immunity against fungal infections in patients with *CARD9* deficiency, including a lower proportion of Th17, impairment of IL-17 production, neutrophils dysfunction, abated responses of the mononuclear phagocyte to fungal-specific stimuli, and dysregulated cytokine production [11]. The proportion of Th17 cells in CARD*9*- deficient patients is a matter of controversy; while Rieber and colleagues revealed the intact production of IL-17 alongside the normal number of Th17 cells and attributed this event to the dispensable role of IL-17 in defense against Aspergillus [5], a decreased number of Th1, Th17, and Th22 was reported in a patient with cutaneous aspergillosis [7]. *CARD9* mutations also impair pro-inflammatory cytokine production following fungal stimulations but not bacterial [11]; this pattern has been observed in patients who have developed aspergillosis [5, 7]. Despite normal counts and function of neutrophils in the peripheral blood of *Aspergillus*-infected patients, the absences of neutrophil infiltration in involved tissue has been observed, probably due to the lack of neutrophil chemo-attractants [5, 8]. Notably, despite recent studies, our patients displayed a reduced number of neutrophils with a normal percentage of CD3+ and CD4+ T cells.

This study provides new insights into the clinical manifestations and a better understanding of the immunopathogenesis of patients harboring CARD9 mutations with aspergillosis.

## Data Availability

The data used in this case report is available upon reques from the corresponding author.
